# Association between shift/night work and irregular periods and period pain among two cohorts of Australian women 16 years apart: findings from the Australian longitudinal study on women’s health

**DOI:** 10.1007/s00420-025-02152-9

**Published:** 2025-06-17

**Authors:** Biresaw Wassihun Alemu, Michael Waller, Leigh R. Tooth

**Affiliations:** Australian Women and Girl’s Health Research Centre, School of Public Health, Faculty of Health, Medicine and Behavioural Sciences, University of Queensland, Brisbane, QLD Australia

**Keywords:** Shift work, Night work, Irregular periods, Period pain, Women's health

## Abstract

**Purpose:**

To examine the associations between shift or night work and irregular periods and period pain among two cohorts of Australian women, using data collected 16 years apart.

**Methods:**

We used data from the 1989–95 (*n* = 6,767) and 1973–78 (*n* = 7,527) cohorts from the Australian Longitudinal Study on Women’s Health, when participants were aged 24–30 years. Logistic regression models were used to assess the association between night or shift work and severe period pain and irregular periods, and to compare them to non-shift or night workers.

**Results:**

Women from the 1989–95 cohort who did night work reported higher odds of having experienced irregular periods (AOR = 1.28, 95% CI: 1.03, 1.59) compared to those who undertook shift work. However, there was no association between night work and severe period pain (AOR = 1.10, 95% CI: 0.86, 1.41). Among women in the 1973–78 cohort, neither severe period pain (AOR = 1.20, 95% CI: 0.82, 1.76) nor irregular periods (AOR = 1.38, 95% CI: 0.92, 2.06) were associated with night work. Across both cohorts, no associations were found between shift or night work and irregular or severe period pain when comparing shift or night workers combined to non-shift or night workers.

**Conclusions:**

Night workers reported higher odds of irregular periods compared to shift workers in the 1989-95 cohort; however, no consistent association was found with severe period pain. Future research should investigate whether this association is causal. Supportive workplace practices may benefit night workers experiencing irregular periods.

**Supplementary Information:**

The online version contains supplementary material available at 10.1007/s00420-025-02152-9.

## Introduction

Regular menstruation is a vital indicator of women’s reproductive well-being, controlled by rhythmic hormonal change (Critchley et al. 2020). Many women of reproductive age experience various menstrual disorders, including irregular periods and period pain, which are among the most common reasons for seeking gynaecological care (Berga 2020). The reported severity of each menstrual disorder among women may vary due to presence of different risk factors including body mass index (BMI), level of stress, number of children, smoking and occupational factors such as night or shift work (Mena et al. 2021; Tang et al. 2020).

Shift work is any arrangement of working hours other than the standard daylight hours. In Australia in 2023, 15% of women participated in shift work (ABS, 2023), while in the European Union around 21% of workers participated in shift work (Alali et al. 2018). The non-standard work arrangement of shift work has been linked with reduced well-being and lower job satisfaction (Bech-Wysocka et al. 2025; Suleiman et al. 2021), and through its impact on job satisfaction, may also impact quality of life (Sánchez-Sellero et al. 2021). Moreover, evidence suggested that job insecurity and instability are linked to increased stress and anxiety, particularly among shift workers, which may lead to lower levels of job satisfaction (Sánchez Sellero et al. 2017; Alghamdi and Bahari 2025).

Shift work has also been found to be a potential risk factor for women’s reproductive health (Hu et al. 2023). Evidence suggests that shift work may disrupt biochemical and neurophysiological functions by disturbing the 24-hour maternal circadian rhythm, which controls the body’s internal clock and affects hormonal cycles (Hu et al. 2023; Chau et al. 2014). Such disruption can interfere with the hypothalamus-pituitary-ovary (HPO) axis, a crucial regulator of reproductive hormones, potentially causing menstrual disorders (Chung et al. 2005; Valsamakis et al. 2019).

Studies suggest a possible association between night or shift work and menstrual disorders (Shim et al. 2024). For instance, a US study of nurses found that those who worked rotating night shifts had a higher likelihood of irregular periods than those who worked regular hours (Lawson et al. 2011). A similar study in Korea also showed associations between shift work and irregular periods (Ok et al. 2019). However, some studies have reported no association between shift work and menstrual disorders (Albert et al. 2016; Moen et al. 2015), and these studies have been limited to specific professions and workplaces, limiting the generalizability of their findings (Albert et al. 2016; Moen et al. 2015). The studies reported above were conducted between 2005 and 2019, reflecting a specific generation (Albert et al. 2016; Moen et al. 2015; Chung et al. 2005; Ok et al. 2019). Given that working conditions have changed over time, the associations between shift/night work on reproductive health may also have varied over time (Razafimahefa et al. 2022). Notably, none of these studies have examined how this association might differ across generations (Albert et al. 2016; Moen et al. 2015; Chung et al. 2005; Ok et al. 2019).

There is also evidence to suggest that women’s menstrual experiences differ from generation to generation due to changes in behavioural and lifestyle factors, sociodemographic shifts, and the evolving nature of work patterns (Li et al. 2023). For example, a US study reported that young women born between 2000 and 2005 experienced menstrual periods earlier than those born between 1950 and 1969 (Wang et al. 2024). Similarly, a study conducted using data from the Australian Longitudinal Study on Women’s Health (ALSWH) found that women born from 1989 to 1995 were more likely to have irregular periods than those born from 1973 to 1978 (Rowlands et al. 2015). However, no research has examined the association of night or shift work with menstrual disorders in two generations.

Understanding generational differences in the associations may inform health and workplace policies to better support menstrual health. Using two cohorts may also strengthen the study’s ability to detect generational differences. The ALSWH offers an opportunity to address this gap. Therefore, the aim of this study is to examine the association between shift or night work and irregular and severe period pain among two cohorts of young Australian women (aged 24–30) who were surveyed 16 years apart, while controlling for relevant covariates.

## Methods

### Study design and participants

This study used data from the ALSWH, which aimed to investigate the health and well-being of Australian women and their access to and use of health services. Since 1996, ALSWH has been collecting surveys from women in three age cohorts born in 1921–26, 1946–51, and 1973–78. In 2013, a new fourth cohort of women born between 1989 and 95 was recruited through a wide range of methods, including social media, and peer referral. The original three cohorts were randomly selected from the national health insurance database (currently known as Medicare) and have been resurveyed at approximately three-year intervals, while the 1989–95 cohort has been surveyed annually or every two years since 2013. The ALSWH received ethical approval from the Human Research Ethics Committees at the University of Queensland and the University of Newcastle in Australia. Informed consent was obtained from all participants before completing each survey. Further information regarding ALSWH methodology has been published (Loxton et al. 2018; Dobson et al. 2015).

The sample for the current study was taken from two cohorts of women born between 1989 and 95 and 1973-78, who had provided information on their menstrual symptoms and work patterns at the time of the survey. We used data from the 2003 survey of the 1973–78 cohort and the 2019 survey of the 1989–95 cohort. These surveys were selected because they included questions on domain-specific work patterns and provided data on women aged 24–30 years. This age range is particularly relevant as it represents a period when most women have typically completed their formal education and have either entered or become more firmly established in the workforce. Additionally, this age range also allowed for a meaningful comparison across cohorts, as these were the first surveys to include participants at equivalent ages. This enabled us to assess how work patterns may be associated with irregular periods and period pain across the two generations. A total of 9,081 women (aged 25–30) in the 1973-78 ALSWH cohort responded to the third survey in 2003. Among these, women who were currently pregnant (*N* = 694) or had missing data on outcomes, exposures, or covariates (*N* = 860) were excluded. From the 1989–95 ALSWH cohort, 8,346 women (aged 24–29) responded to the sixth survey in 2019. In this group, women who were currently pregnant (*n* = 361) and those with missing data on outcomes, exposures, or covariates (*n* = 1,218) were excluded. The final sample included in the analysis consisted of 7,527 participants from the 1973–78 cohort’s third survey and 6,767 participants from the 1989–95 cohort’s sixth survey (Figure S1 and S2).

### Measurement of menstrual symptoms

Women were asked about their menstrual symptoms. At each survey, two separate questions were asked: “In the last 12 months, have you experienced [severe period pain; irregular periods]?“. The possible responses were measured using a 4-category Likert scale (never, rarely, sometimes, or often).Women were defined as consistently having the symptom if their response was ‘often’, and as not consistently having the symptom if their response was ‘sometimes’, ‘rarely’ or ‘never’. We selected “often” threshold to capture those women who experienced severe or persistent symptoms, consistent with the categories used in previous studies using the ALSWH cohorts (Gete et al. 2024; Jin et al. 2025).

### Measurement of work patterns

Participants were asked whether their work involved shift work, night work, casual work, working from home, self-employment, or holding more than one job (categories were not mutually exclusive). They were also asked if they were not in paid work. The work pattern variable was then categorized into three groups: “shift or night” for participants who reported working shifts or night shifts; “not shift or night” for participants who worked but not in shift or night work; and “not paid work” for participants who reported not being in paid work.

### Assessment of covariates

The study included a range of covariates that were identified a priori as having a potential association with both irregular periods, period pain and shift or night work (Barcikowska et al. 2020; Shim et al. 2024; Zhao et al. 2012). These were the socio-demographic characteristics marital status (single, defacto or married, and divorced/widowed/separated), and the highest level of education (up to grade 12, trade or certificate or diploma, university degree/higher degree). Occupation was also available in the dataset but was not included as a covariate due to its correlation with the work pattern outcome.

Health risk characteristics included BMI and stress. BMI was classified as underweight (BMI < 18.5 kg/m²), normal weight (BMI 18.5 to 24.9 kg/m²), overweight (BMI 25 to 29.9 kg/m²), and obese (BMI ≥ 30 kg/m²). The level of stress among study participants was assessed using the Perceived Stress Questionnaire, which has been developed and validated for the ALSWH study. The tool examined the level of perceived stress in 11 life domains in specific areas of life, including study, relationships, motherhood /children, employment and own health Participants rated their stress levels in each domain using a five-point scale: not at all stressed, somewhat stressed, moderately stressed, very stressed, and extremely stressed. A mean stress score was calculated for each participant, ranging from 0 to 4, with higher scores indicating greater perceived stress. Based on these scores, stress levels were categorised as not or somewhat stressed (0–0.99), moderately stressed (1–1.99), and very or extremely stressed (2–4) (Bell and Lee 2002). Finally, number of children was captured by the number of live children the women had given birth to (never had children, one child, and two or more children).

### Statistical analysis

Descriptive statistics were used to compare and describe socio-demographic and health risk characteristics of women. In the first stage, we compared women engaged in shift or night work with those who worked but did not do shift or night work and those not in paid work. In the second stage, we compared night workers to shift workers separately. Logistic regression was used to examine associations separately between work patterns and irregular periods and severe period pain. The results were reported using odds ratios (ORs) and 95% confidence intervals (CIs). The analyses for the logistic regression models were carried out in separate models for irregular periods and severe period pain. A crude model without covariates was run first (Model 1). Covariates were added in steps: Model 2 added educational status and marital status; Model 3 added BMI and stress; Model 4 added number of children. Data analyses were conducted using Stata software (version 18.0; Stata Corp). The final sample included in the analysis consisted of 7,527 participants from the 1973–78 cohort’s third survey and 6,767 participants from the 1989–95 cohort’s sixth survey. This sample provide sufficient statistical power to detect significant associations between shift work and irregular periods, and severe period pain.

## Results

### Study participant characteristics

Tables 1 and 2 provide an overview of the participants’ socio-demographic characteristics, health risk factors and number of children, and the occurrence of irregular periods and severe period pain. The 1973–78 cohort were a mean age of 27 years (SD ± 1.4), while the 1989–95 cohort were a mean age of 26 years (± 1.8).

**Table 1:** Socio-demographic, health risk factors and number of children of participants in the 1973–78 ALSWH cohort (*n* = 9,081) at survey 3 in 2003, and of participants in this analysis (*n* = 7,527)

Women’s characteristics	All women (*n* = 9,081) ^column %^	Irregular periods (*n* = 7,527), row %			Period pain (*n* = 7,527), row %		
		Never/rarely/sometimes	Often	*P* ^a^ Value	Never/rarely/sometimes	Often	*P* ^a^ Value
All women		6,835 (90.8)	692 (9.2)		6,814 (90.5)	713 (9.5)	
Women’s age(y), mean (SD)	27 (1.4)	27 (1.4)	27 (1.5)	0.83	27 (1.4)	27 (1.5)	0.03
Marital status, n (%) ^m^				0.62			0.37
Single	3,157 (34.8)	2,567 (91.1)	251 (8.9)		2,537 (90.0)	281 (10.0)	
Married or de facto	5,549 (61.1)	4,014 (90.7)	411 (9.3)		4,023 (90.7)	402 (9.1)	
Separated/widowed/divorced	339 (3.7)	254 (89.4)	30 (10.6)		254 (89.3)	30 (10.6)	
Educational status n (%) ^m^				< 0.001			< 0.001
Less than high school	934 (10.3)	654 (87.9)	90 (12.1)		666 (89.5)	78 (10.5)	
High school/trade certificate/diploma	4,002 (44.1)	2,999 (89.9)	338 (10.1)		2,970 (89.0)	365 (11.0)	
Degree or higher	3,931 (43.3)	3,182 (92.3)	264 (7.7)		3,178 (92.2)	268 (7.8)	
Work pattern				< 0.001			0.99
Shift or night worker	1,518 (16.7)	1,217 (91.2)	117 (8.8)		1,208 (90.5)	126 (9.5)	
Not shift or night work	5,554 (61.2)	4,449 (91.5)	415 (8.5)		4,404 (90.4)	460 (9.6)	
No paid work	1,646 (18.1)	1,169 (87.9)	160 (12.1)		1,202 (90.5)	127 (9.5)	
Body mass index, n (%)				< 0.001			< 0.001
Underweight	363 (4.0)	289 (87.6)	41 (12.4)		283 (85.8)	47 (14.2)	
Healthy weight	4,732 (52.1)	4,044 (92.1)	349 (7.9)		4,018 (91.5)	375 (8.5)	
Overweight	1,792 (19.7)	1,515 (90.9)	150 (9.1)		1,495 (89.8)	170 (10.2)	
Obese	1,217 (13.4)	987 (86.6)	152 (13.4)		1,018 (89.4)	121 (10.6)	
Number of children, n (%)				< 0.001			< 0.001
0	6,164 (67.9)	4,747 (91.8)	425 (8.2)		4,704 (89.8)	533 (10.2)	
1	1,444 (15.9)	946 (89.9)	106 (10.1)		998 (93.4)	71 (6.6)	
≥2	1,473 (16.2)	1,049 (87.2)	154 (12.8)		1,112 (91.1)	109 (8.9)	
Stress, n (%) ^m^				< 0.001			< 0.001
Not/somewhat stressed	4,525 (49.8)	3,375 (93.4)	239 (6.6)		3,436 (93.4)	236 (6.4)	
Moderately/stressed	3,769 (41.5)	2,832 (89.3)	339 (10.7)		2,861 (89.2)	348 (10.8)	
Very/extremely stressed	724 (8.0)	535 (83.3)	107 (16.7)		517 (80.0)	129 (20.0)	

Key: P^a^ values were determined using Pearson chi-square tests or t tests; Values are presented as both column and row percentage (%). ^**m**^ Missing values: marital status, *n* = 36; educational status, *n* = 214; BMI, *n* = 977, work pattern, *n* = 363; stress, *n* = 63

**Table 2:** Socio-demographic, health risk factors and number of children of participants in the 1989-95 ALSWH cohort(*n* = 8,346) at survey 6 in 2019, and of participants in this analysis (*n* = 6,767)

Women’s characteristics	All women (*n* = 8,346) ^column %^	Irregular period (= *n* = 6,767), row %			Period pain (*n* = 6,767), row %		
		Never/rare/ sometimes	Often	*P* ^a^ Value	Never/rare/ sometimes	Often	*P* ^a^ Value
All women		5,405 (79.9)	1362 (20.1)		5,754 (85.0)	1,013 (15.0)	
Women’s age (y), mean (SD)	26 (1.8)	26 (1.8)	26 (1.8)	< 0.001	26 (1.8)	26 (1.8)	< 0.001
Marital status, n (%) ^m^				0.10			
Single	3,785 (45.3)	2,741 (80.3)	672 (19.7)		2,937 (86.1)	476 (13.9)	0.04
Married or de facto	4,076 (48.8)	2,614 (79.6)	669 (20.4)		2,755 (83.9)	528 (16.1)	
Separated/widowed/divorced	79 (0.9)	50 (70.4)	21 (29.6)		62 (87.3)	9 (12.7)	
Educational status n (%)^m^				< 0.001			< 0.001
Less than high school	172 (2.1)	86 (64.7)	47 (35.3)		94 (70.7)	39 (39.7)	
High school/trade certificate/diploma	2,421 (29.0)	1,463 (75.1)	485 (24.9)		1,551 (75.6)	398 (24.4)	
Degree or higher	5,351 (64.1)	3,855 (82.3)	830 (17.7)		4,109 (87.7)	576 (12.3)	
Work pattern				< 0.001			0.98
Shift or night worker	2,245 (26.9)	1,657 (78.9)	444 (21.1)		1,783 (84.7)	321 (15.3)	
Not shift or night work	4,407 (52.8)	3,341 (81.1)	777 (18.9)		3,545 (86.1)	574 (13.9)	
No paid work	1 612 (7.3)	403 (74.1)	141 (25.9)		426 (78.3)	118 (21.7)	
Body mass index, n (%)				< 0.001			< 0.001
Underweight	218 (2.6)	134 (74.9)	45 (25.1)		157 (87.7)	22 (12.3)	
Healthy weight	3,819 (45.8)	2,806 (82.7)	588 (17.3)		2,969 (87.5)	425 (12.5)	
Overweight	1,964 (23.5)	1,384 (81.2)	320 (18.8)		1,440 (84.5)	264 (15.5)	
Obese	1,759 (21.1)	1,081 (72.5)	409 (27.4)		1,188 (79.7)	302 (20.3)	
Number of children, n(%)				< 0.001			0.05
0	7,042 (84.4)	4,851 (80.6)	1,167 (19.4)		5,138 (85.4)	880 (14.6)	
1	755 (9.0)	276 (73.2)	101 (26.8)		314 (83.3)	63 (16.7)	
≥2	549 (6.6)	278 (74.7)	94 (25.3)		302 (81.2)	70 (18.8)	
Stress, n (%) ^m^				< 0.001			< 0.001
Not/somewhat stressed	3,351 (40.1)	2,353 (84.5)	431 (15.5)		2,508 (89.9)	279 (10.1)	
Moderately/stressed	3,677 (44.1)	2,488 (78.7)	673 (21.3)		2,665 (84.3)	497 (15.7)	
Very/extremely stressed	982 (11.8)	560 (68.5)	258 (31.5)		581 (71.0)	237 (28.9)	

Key: *P* values were determined using Pearson chi-square tests or *t* tests; Values are presented as both column and row percentage (%). ^**m**^ Missing values: area of residence, *n* = 306; marital status, *n* = 406; educational status, *n* = 402; BMI, *n* = 586, work pattern, *n* = 1.082; stress, *n* = 336

In the 1989-95 cohort, the prevalence of irregular periods was double the rate reported by the 1973-78 cohort (20.1% vs. 9.2%) and reporting of period pain was also more common in the women born 1989-95 (15.0% vs. 9.5%) (Tables 1 and 2).

In the 1973–78 cohort, irregular periods were more common among women who were not in paid work, had lower educational attainment, or were either obese or underweight. Women with more than two children and those experiencing high stress levels also frequently reported irregular menstruation. Similarly, period pain was more common among women without children and those with high stress. No association was found between work patterns and period pain, and marital status showed no significant association with either period pain or irregular periods in this cohort (Table 1).

We observed similar trends in the 1989–95 cohort. Irregular periods were more common among women who were not in paid work, divorced, widowed or separated, had lower educational attainment, or were either obese or underweight. Women who reported high stress levels were more likely to experience irregular periods. Similarly, period pain was more common among women who were not in paid work, had lower educational attainment, more than two children, or those who were obese or more stressed (Table 2).

### Association of shift or night work with irregular periods and period pain

Tables 3 and show the analyses of the associations between shift or night work with irregular periods and period pain in the 1973-78 and 1989-95 cohorts. The association between shift or night work and menstrual symptoms varied depending on the type of work patterns. In the 1973–78 cohort, univariate analysis indicated that women not in paid work had higher odds of having experienced irregular periods compared to those who worked but not in shift or night work (Odds Ratio [OR] = 1.47, 95% CI: 1.21, 1.78). However, this association was no longer significant after controlling for covariates (Adjusted Odds Ratio [AOR] = 1.13, 95% CI: 0.90, 1.41). Similarly, women who participated in night work were more likely to have experienced irregular periods compared to shift workers in the unadjusted model (OR = 1.49, 95% CI: 1.00, 2.21), but not in the fully adjusted model (AOR = 1.38, 95% CI: 0.92, 2.06). No significant association was found between women who did shift, or night work and period pain compared to those who worked but not in shift or night work (unadjusted OR = 0.99, 95% CI: 0.81, 1.23; AOR = 0.98, 95% CI: 0.79, 1.21) **(Table 3).**

**Table 3:** Associations between shift or night work and irregular periods and period pain among the 1973-78 ALSWH cohort (*n* = 7,527)

Variables	Irregular periods Often vs. never/rarely/sometimes				Period pain Often vs. never/rarely/sometimes			
Work pattern	Model 1, OR (95% CI)	Model 2, AOR (95% CI)	Model 3, AOR (95% CI)	Model 4, AOR (95% CI)	Model 1, OR (95% CI)	Model 2, AOR (95% CI)	Model 3, AOR (95% CI)	Model 4, AOR (95% CI)
Shift or night	1.03 (0.83, 1.28)	1.02 (0.83–1.27)	1.03 (0.83,1.27)	1.02 (0.82,1.27)	0.99 (0.81,1.23)	0.98 (0.80,1.22)	0.99 (0.80,1.22)	0.98 (0.79,1.21)
No paid work	1.47 (1.21, 1.78)*	1.33 (1.09,1.63)*	1.25 (1.02, 1.54)*	1.13 (0.90,1.41)	1.01 (0.82, 1.24)	0.93 (0.75,1.15)	0.87 (0.70,1.09)	1.06 (0.84,1.35)
Not shift or night	1.00	1.00	1.00	1.00	1.00	1.00	1.00	1.00
Night workers	1.49 (1.00, 2.21)*	1.51 (1.01, 2.24)*	1.38 (0.93, 2.07)	1.38 (0.92, 2.06)	1.25 (0.87, 1.83)	1.24 (0.85,1.81)	1.16 (0.79,1.69)	1.20 (0.82,1.76)
Shift workers	1.00	1.00	1.00	1.00	1.00	1.00	1.00	1.00

Abbreviations: OR odds ratio; AOR, adjusted odds ratio; CI, confidence interval; * indicates statistical significance (*p* < 0.05). 1.00, reference group

Model 1: Unadjusted, Model 2: model 1 plus education level and marital status, Model 3: model 2 plus BMI and stress, Model 4: model 3 plus number of children

In the 1989–95 cohort, univariate analysis indicated that women not in paid work had higher odds of having experienced irregular periods compared to those who worked but not in shift or night work (OR = 1.50, 95% CI: 1.22,1.85), but this association was no longer significant after controlling for covariates (AOR = 1.07, 95% CI: 0.85,1.35) (Table 4). Similarly, women who participated in shift or night work were more likely to have experienced irregular periods compared to those who worked but not in shift or night work in the unadjusted model (OR = 1.15, 95% CI: 1.01,1.31), but not in the fully adjusted model (AOR = 1.11, 95% CI: 0.96,1.26). Women who worked nights reported higher odds of having experienced irregular periods compared to other shift workers (unadjusted OR = 1.24, 95% CI: 1.00, 1.53; AOR = 1.28, 95% CI: 1.03, 1.59).

The odds of reporting period pain were higher among women not in paid work in the unadjusted model (OR = 1.71, 95% CI: 1.37, 2.14). After adjusting for all covariates, there was no longer an association between no paid work and period pain compared to non-shift or night work (AOR = 1.21, 95% CI: 0.95, 1.55). We found no significant association between night work compared with shift work and period pain in either model (unadjusted OR = 1.07, 95% CI: 0.85, 1.37; AOR = 1.10, 95% CI: 0.86, 1.41) (Table 4). 

**Table 4:** Associations between shift or night work and irregular periods and period painamong the 1989-95 ALSWHcohort (*n* = 6,767)

Variables	Irregular periods Often vs. never/rarely/sometimes				Period pain Often vs. never/rarely/sometimes			
Work pattern	Model 1, OR (95% CI)	Model 2, AOR (95% CI)	Model 3, AOR (95% CI)	Model 4, AOR (95% CI)	Model 1, OR (95% CI)	Model 2, AOR (95% CI)	Model 3, AOR (95% CI)	Model 4, AOR (95% CI)
Shift or night	1.15 (1.01, 1.31)*	1.12 (0.98, 1.27)	1.10 (0.96, 1.26)	1.11 (0.96,1.26)	1.11 (0.96,1.29)	1.07 (0.92,1.25)	1.05 (0.90,1.22)	1.05 (0.89,1.23)
No paid work	1.50 (1.22, 1.85)*	1.23 (0.99,1.53)	1.10 (0.88, 1.37)	1.07 (0.85,1.35)	1.71 (1.37,2.14)*	1.36 (1.07,1.71)*	1.16 (0.92,1.48)	1.21 (0.95, 1.55)
Not shift or night	1.00	1.00	1.00	1.00	1.00	1.00	1.00	1.00
Night workers	1.24 (1.00,1.53)*	1.28 (1.03,1.58)*	1.27 (1.03, 1.59)*	1.28 (1.03,1.59)*	1.07 (0.85, 1.37)	1.13 (0.88,1.44)	1.11 (0.87,1.42)	1.21 (0.95, 1.55)
Shift workers	1.00	1.00	1.00	1.00	1.00	1.00	1.00	1.00

Abbreviations: OR odds ratio; AOR, adjusted odds ratio; CI, confidence interval; * indicates statistical significance (*p* < 0.05). 1.00, reference group

Model 1: Unadjusted, Model 2: model 1 plus education level and marital status, Model 3: model 2 plus BMI and stress, Model 4: model 3 plus number of children

### Covariates associated with irregular periods and severe period pain

Supplementary Tables S1 and S2 show associations between the covariates included in this study and irregular periods and severe period pain. We examined the role these covariates played in the associations between shift or night work and irregular periods and period pain after noticing that significant associations in univariate analyses routinely became non-significant after adjustment. In this study, we found important covariates associated with irregular periods and severe period pain, indicating both consistent patterns and generational differences between the 1973–78 and 1989–95 cohorts. Women who experienced high levels of psychological stress reported higher odds of both severe period pain and irregular periods in both cohorts. This highlights the importance of psychological stress to menstrual health. BMI was also an important factor, with both underweight and obese women being more likely to experience menstrual irregularities and severe period pain compared to women of healthy weight. This emphasises how crucial weight control is for women who are of reproductive age. Level of educational attainment also showed a consistent association, with women with lower levels of education being more likely to report both irregular periods and severe period pain.

There were some variations between the cohorts despite this similarity. For instance, in the 1973–78 cohort, women with two or more children were more likely to report irregular periods but less likely to experience severe period pain, however the same pattern was not observed in the 1989–95 cohort (Tables S1 and S2).

## Discussion

This study examined the association between shift or night work and irregular periods and severe period pain using data from two national cohorts of women within the same age range, using similar measures. This method provides an initial step towards understanding this important but often overlooked aspect of women’s health.

The present study indicates that night workers reported higher odds of often experiencing irregular periods compared to shift workers in the 1989-95 cohort. These associations were independent of potential covariates, including socio-demographic factors and health risk and the number of children. However, no significant association was found among women in 1973-78 cohort once covariates were adjusted for. The observed differences between the two cohorts may be due to disparities in the reported prevalence of irregular periods, with a higher prevalence of irregular periods (20.1% vs. 9.2%) reported in the 1989–95 cohort than the 1973–78 cohort, which increased the power to detect a difference. Evidence suggests that increased awareness of menstrual health and differences in social contexts may also affect the association between shift work and irregular periods across the two cohorts. The 1989-95 cohort grew up during a period of rapid technological advancement and high levels of interpersonal connectivity via mobile phones and social media (Loxton et al. 2018). The expansion of the internet and the rise of digital health resources access may have increased women’s awareness of menstrual health (Cunningham et al. 2024; Armour et al. 2021). Additionally, societal attitudes toward menstruation have changed overtime (Peranovic and Bentley 2017). Evidence has shown that in older generations, and in some cultures, menstruation was often considered taboo, and women might have felt too ashamed to disclose their menstrual symptoms. However, in young generations increased awareness of menstrual health has contributed to a shift toward normalizing menstruation as a natural biological process, which may encourage more women in recent generations to report menstrual irregularities (Peranovic and Bentley 2017; DeMaria et al. 2020). Further research is needed to explore these differences by examining detailed work schedules, types of work, and occupational settings across cohorts. Across both cohorts, no associations were found between shift or night work and irregular or severe period pain when comparing shift or night workers combined to non-shift or night workers once covariates were adjusted for.

### Comparison with other studies

Night workers reported higher odds of irregular periods compared to shift workers in the 1989-95 cohort. Our finding is consistent with a previous Australian study, which reported that night shift workers were more likely than day workers to have menstrual irregularity (Fernandez et al. 2020). In line with our findings, a study conducted among Korean workers found that night workers reported higher odds of irregular periods than shift workers (Kim et al. 2024). Likewise, studies conducted among health care providers in China, Japan, and Taiwan have indicated that night shift work is linked to higher odds of irregular periods, compared to those on rotating shifts (Wang et al. 2016; Su et al. 2008; Mayama et al. 2020). However, these studies included broader age groups, making direct comparisons with our study challenging. The increased odds of irregular periods observed in night workers may be justified by several mechanisms including disruption in circadian rhythms, light-induced melatonin suppression and prolonged sleep disturbances. Circadian rhythm plays important role in the synchronization of hormonal signals governing the menstrual cycle, and disruption to this rhythm may result in menstrual irregularity (Boivin et al. 2022; Kim et al. 2024). Evidence showed that poor sleep quality often linked to night work can result in sleep deprivation, which disrupts hormone regulation and may contribute to menstrual irregularity (Baker and Driver 2007). Exposure to light during night shift work can interfere with the pineal gland’s normal secretion of melatonin, a hormone that regulates sleep and affects other body functions. Melatonin production normally peaks at night and plays a role in regulating the menstrual cycle. Light exposure at night causes a decrease in melatonin secretion, which may further disrupt physiological functions and may lead to menstrual irregularities (Cable et al. 2021; Miguet et al. 2020; Kozaki et al. 2015).

Our research revealed no significant association between shift or night work and irregular periods compared to workers who do not engage in such shifts or who are not in paid work, once covariates were adjusted for. However, research conducted in Korea indicated that the likelihood of experiencing menstrual irregularities is greater among women who work shifts than those who have fixed work schedules (Ok et al. 2019). Likewise, research conducted in the US indicated that the higher odds of experiencing irregular periods were reported among women who work rotating shifts than those who do not (Lawson et al. 2011). In that study, Lawson et al. (2011) reported that irregular periods were higher among women who worked longer durations. Both the Ok et al.(2019) and Lawson et al.(2011) studies included women aged 25 to 50 years. However, we included women aged 30 years or younger. As women age, particularly during the menopausal transition, the prevalence of irregular periods tends to increase (Mackey 2009). This disparity in age groups may have a substantial impact on the experiences of irregular periods.

In our study no significant association was observed between shift or night work and severe period pain when compared to non-shift or night workers or women not in paid work. However, a study conducted in Chinese nurses reported that the risk of period pain was higher among women with shift work schedules than those working fixed day shifts (Wang et al. 2016). Similarly, a study conducted in Egypt found that nurses working shifts reported higher odds of period pain compared to those working standard hours (Mirfat and Mageda 2016), although this study did not control for important covariates strongly linked with severe period pain.

### Strengths and limitations

The strength of this study was the use of data from a nationally representative sample of young women. We included women from two cohorts, 16 years apart, which provides valuable insights into the association between night or shift work and irregular periods and severe period pain in two generational cohorts. In our statistical analysis we also controlled for potential covariates such as socio-demographic factors, health risks, and the number of children. However, this study has some limitations. First, the questions we used to capture information on menstrual symptoms and night or shift work were collected retrospectively, introducing the potential for recall bias. The question also did not specifically define ‘irregular’. Second, this study comprised women within the age range of 24–30 years, limiting the generalizability of our findings to women beyond this age range. Third, participants in the ALSWH 1989-95 and 1973–78 cohorts were predominantly from higher socio-economic backgrounds, which limits the generalizability of the findings to broader populations (Dobson et al. 2015). Furthermore, as the study only examines the associations cross-sectionally we cannot infer causality.

## Conclusion

Findings from our study suggest that night workers reported higher odds of irregular periods compared to shift workers in the 1989-95 cohort. However, no significant association was found among women born 1973-78. The possible reason might be due to the higher reported prevalence of irregular periods in the 1989-95 cohort. Increased awareness of menstrual health among younger generations and change in socio-cultural attitude to women’s menstrual health might also contribute to this generational difference. In both cohorts, no significant associations were found for either severe period pain or irregular periods, when comparing shift or night workers with non-shift or night workers or women not in paid work. The results of this study suggest that work patterns may be differentially associated with specific menstrual symptoms, with night workers reporting higher odds of irregular periods compared to shift workers. Overall, our findings revealed insights into the association between night or shift work and irregular periods and severe period pain. Future research should investigate whether this association is causal.

## Electronic supplementary material

Below is the link to the electronic supplementary material.

Supplementary Material 1: Table S1. Factors associated with irregular periods and period pain among the 1973-78 ALSWH cohort (*n* = 7,527). Table S2. Factors associated with irregular periods and period pain among 1989-95 ALSWH cohort (*n* = 6,767).

Supplementary Material 2: Fig. S1: Flow charts for inclusion of participants from the 1973-78 cohorts. Fig. S2: Flow charts for inclusion of participants from the 1989-95 cohorts

## Data Availability

ALSWH survey data are owned by the Australian Government Department of Health and Aged Care and due to the personal nature of the data collected, release by ALSWH is subject to strict contractual and ethical restrictions. De-identified data are available to collaborating researchers where a formal request to make use of the material has been approved by the ALSWH Data Access Committee. The committee is receptive of requests for datasets required to replicate results. Information on applying for ALSWH data is available from https://alswh.org.au/for-data-users/applying-for-data/.
