# Correction: Association between shift/night work and irregular periods and period pain among two cohorts of Australian women 16 years apart: findings from the Australian longitudinal study on women’s health

**DOI:** 10.1007/s00420-025-02156-5

**Published:** 2025-07-03

**Authors:** Biresaw Wassihun, Alemu Michael Waller, Leigh R. Tooth

**Affiliations:** Australian Women and Girl’s Health Research Centre, School of Public Health, Faculty of Health, Medicine and Behavioural Sciences, University of Queensland, Brisbane, QLD Australia

**Correction to: International Archives of Occupational and Environmental Health** 10.1007/s00420-025-02152-9

In Table 4 of this article, the data were mistakenly presented in text format but should have been in table format. The correct version is given below.

**Table 4:** Associations between shift or night work and irregular periods and period pain among the 1989–95 ALSWH cohort (*n* = 6767)

Variables	Irregular periods Often vs. never/rarely/ sometimes				Period pain Often vs. never/rarely/ sometimes			
Work pattern	Model 1, OR (95% CI)	Model 2, AOR (95% CI)	Model 3, AOR (95% CI)	Model 4, AOR (95% CI)	Model 1, OR (95% CI)	Model 2, AOR (95% CI)	Model 3, AOR (95% CI)	Model 4, AOR (95% CI)
Shift or night	1.15 (1.01, 1.31)*	1.12 (0.98, 1.27)	1.10 (0.96, 1.26)	1.11 (0.96,1.26)	1.11 (0.96,1.29)	1.07 (0.92,1.25)	1.05 (0.90,1.22)	1.05 (0.89,1.23)
No paid work	1.50 (1.22, 1.85)*	1.23 (0.99,1.53)	1.10 (0.88, 1.37)	1.07 (0.85,1.35)	1.71 (1.37,2.14)*	1.36 (1.07,1.71)*	1.16 (0.92,1.48)	1.21 (0.95, 1.55)
Not shift or night	1.00	1.00	1.00	1.00	1.00	1.00	1.00	1.00
Night workers	1.24 (1.00,1.53)*	1.28 (1.03,1.58)*	1.27 (1.03, 1.59)*	1.28 (1.03,1.59)*	1.07 (0.85, 1.37)	1.13 (0.88,1.44)	1.11 (0.87,1.42)	1.10 (0.86,1.41)
Shift workers	1.00	1.00	1.00	1.00	1.00	1.00	1.00	1.00

Model 1: Unadjusted, Model 2: model 1 plus education level and marital status, Model 3: model 2 plus BMI and stress, Model 4: model 3 plus number of children.

*OR* Odds ratio, *AOR* Adjusted odds ratio, *CI* Confidence interval.

*Indicates statistical significance (*p* < 0.05). 1.00 reference group.

 The original article has been corrected.

