# Supplementary material for: Association between shift/night work and irregular periods and period pain among two cohorts of Australian women 16 years apart: findings from the Australian longitudinal study on women’s health
Source: Int Arch Occup Environ Health. 2025 Jun 17;98(6):537–47. doi: 10.1007/s00420-025-02152-9 (PMC12331763; doi:10.1007/s00420-025-02152-9)
Supplement: Supplementary file 1 — Supplementary Material 1: Table S1. Factors associated with irregular periods and period pain among the 1973-78 ALSWH cohort (n = 7,527). Table S2. Factors associated with irregular periods and period pain among 1989-95 ALSWH cohort (n = 6,767). [file 420_2025_2152_MOESM1_ESM.docx]

**Table S1**. Factors associated with irregular periods and period pain among the 1973-78 ALSWH cohort (n=7,527)

| Variables | **Irregular periods**  Often vs never/rarely/sometimes | **Severe period pain**  Often vs never/rarely/ sometimes |
| --- | --- | --- |
| Work pattern | AOR (95% CI) | AOR (95% CI) |
| Shift/night work | 1.02 (0.82,1.27) | 0.98 (0.79,1.21) |
| No paid work | 1.13 (0.90,1.41) | 1.06 (0.84,1.35) |
| Not shift/night work | 1.00 | 1.00 |
| Marital status |  |  |
| Married or de facto | 1.00 | 1.00 |
| Single | 0.99 (0.82,1.19) | 0.85 (0.72, 1.02) |
| Separated/widowed/Divorced | 0.84 (0.56,1.25) | 0.86 (0.58, 1.29) |
| Educational status |  |  |
| Less than high school | 1.24 (0.94,1.64) | 1.55 (1.16, 2.07)* |
| High school/trade certificate/ diploma | 1.16 (0.97, 1.39) | 1.54 (1.29,1.83)* |
| Degree or higher | 1.00 | 1.00 |
| Body mass index |  |  |
| Healthy weight | 1.00 | 1.00 |
| Underweight | 1.55 (1.09,2.19)* | 1.68 (1.20, 2.34)* |
| Overweight | 1.04 (0.85, 1.27) | 1.17 (0.96, 1.42) |
| Obese | 1.51 (1.22,1.86)* | 1.13 (0.90,1.41) |
| Number of children |  |  |
| 0 | 1.00 | 1.00 |
| 1 | 1.13 (0.87,1.44) | 0.52 (0.39, 0.68)* |
| ≥2 | 1.39 (1.09,1.79)* | 0.64 (0.49,0.83)* |
| Stress |  |  |
| Not/somewhat stressed | 1.00 | 1.00 |
| Moderately/stressed | 1.64 (1.38,1.95)* | 1.79 (1.50, 2.13)* |
| Very/extremely stressed | 2.65 (2.06,3.41)* | 3.57 (2.80, 4.54)* |

Note: Results are from logistic regression models. AOR, adjusted odds ratio; CI, confidence interval; * indicates statistical significance (*p* < 0.05). The covariate estimates are derived from the model comparing shift/night work and no paid work to non-shift or non-night work.

**Table S2.** Factors associated with irregular periods and period pain among 1989-95 ALSWH cohort (n=6,767)

| Variables | **Irregular periods**  Often vs never/rarely/ sometimes | **Period pain**  Often vs never/rarely/ sometimes |
| --- | --- | --- |
| Work pattern | AOR (95% CI) | AOR (95% CI) |
| Shift/night work | 1.11(0.96,1.26) | 1.05 (0.89,1.23) |
| No paid work | 1.07 (0.85,1.35) | 1.21 (0.95, 1.55) |
| Not shift/night | 1.00 | 1.00 |
| Marital status |  |  |
| Married or de facto | 1.00 | 1.00 |
| Single | 0.91 (0.80,1.03) | 0.76 (0.66, 0.87)* |
| Separated/widowed/Divorced | 1.13 (0.66,1.94) | 0.43 (0.20, 0.88)* |
| Educational status |  |  |
| Less than high school | 1.90 (1.29,2.79)* | 2.33 (1.55, 3.51)* |
| High school/trade certificate/ diploma | 1.26 (1.10,1.45)* | 1.52 (1.31,1.77)* |
| Degree or higher | 1.00 | 1.00 |
| Body mass index |  |  |
| Healthy weight | 1.00 | 1.00 |
| Underweight | 1.51 (1.06,2.15)* | 0.89 (0.56, 1.42) |
| Overweight | 1.03 (0.88, 1.20) | 1.16 (0.98, 1.38) |
| Obese | 1.50 (1.29,1.75)* | 1.38 (1.16,1.64)* |
| Number of children |  |  |
| 0 | 1.00 | 1.00 |
| 1 | 1.20 (0.93,1.55) | 0.83 (0.61, 1.11) |
| ≥2 | 1.06 (0.81,1.39) | 1.86 (0.64,1.16) |
| Stress |  |  |
| Not/somewhat stressed | 1.00 | 1.00 |
| Moderately/stressed | 1.38 (1.21,1.58)* | 1.58 (1.35, 1.86)* |
| Very/extremely stressed | 2.17 (1.79,2.62)* | 3.26 (2.65, 3.99)* |

Note: Results are from logistic regression models. AOR, adjusted odds ratio; CI, confidence interval; * indicates statistical significance (*p* < 0.05). The covariate estimates are derived from the model comparing shift/night work and no paid work to non-shift or non-night work.

Supplementary Table S3. Occupational status of study participants.

| **1973-78 cohort** | Work pattern | (n=7,353) |  |
| --- | --- | --- | --- |
| Occupation | Shift or night | Not shift or night | No paid work |
| Manager or professional | 757 (58) | 2,545 (53.6) | 69 (5.3) |
| Clerical or Sales | 374 (28.7) | 1,698 (35.8) | 64 (4.9) |
| Trades, Transport or labourer | 155 (11.9) | 321 (6.8) | 10 (0.8) |
| No paid job | 19 (1.4) | 181(3.8) | 1,160(89.0) |
| Total | 1305 | 4705 | 1303 |
|  |  |  |  |
| **1989-95 cohort** | Work pattern | (n= 6,762) |  |
| Occupation | Shift or night | Not shift or night | No paid work |
| Manager or professional | 1,358 (64.6) | 2,781 (67.5) | 31(5.7) |
| Clerical or Sales | 572 (27.2) | 1,023 (24.8) | 15(2.8) |
| Trades, Transport or labourer | 141 (6.7) | 171 (4.2) | 6(1.1) |
| No paid job | 30(1.4) | 142(3.4) | 492 (90.4)) |
| Total | 2,101 | 4,117 | 544 |
