# Supplementary material for: Association between shift/night work and irregular periods and period pain among two cohorts of Australian women 16 years apart: findings from the Australian longitudinal study on women’s health
Source: Int Arch Occup Environ Health. 2025 Jun 17;98(6):537–47. doi: 10.1007/s00420-025-02152-9 (PMC12331763; doi:10.1007/s00420-025-02152-9)
Supplement: Supplementary file 2 — Supplementary Material 2: Fig. S1: Flow charts for inclusion of participants from the 1973-78 cohorts. Fig. S2: Flow charts for inclusion of participants from the 1989-95 cohorts [file 420_2025_2152_MOESM2_ESM.docx]

**1973-78 Cohort**

Women aged 18-23 years recruited in 1996 (n=14,247)

Lost to follow-up between Surveys 1 and 3 (n=5,166)

Women who completed Survey 3 in 2000, aged 25-30 years (n=9,081)

Excluded because of current pregnancy (n=694)

Missing data (n=860):

Outcome (n=342), Exposure (n=22)

Covariates (n=496)

Final study sample

(n=7,527)

**Figure S1**. Flow charts for inclusion of participants from the 1973-78 cohorts.

**1989-95 Cohort**

Women aged 18-23 years recruited in 2013 (n=17,010)

Lost to follow-up between Surveys 1 and 6 (n=8,664)

Women who completed Survey 6 in 2019, aged 24-29 years (n= 8,346)

Excluded because of current pregnancy (n=361)

Missing data (n=1,218):

Outcome (n=1,016), Exposure (n=2), Covariates (n=200)

Final study sample

(n=6,767)

**Figure S2**. Flow charts for inclusion of participants from the 1989-95 cohorts.
